# Differential gene network analysis for the identification of asthma-associated therapeutic targets in allergen-specific T-helper memory responses

**DOI:** 10.1186/s12920-016-0171-z

**Published:** 2016-02-27

**Authors:** Niamh M. Troy, Elysia M. Hollams, Patrick G. Holt, Anthony Bosco

**Affiliations:** Telethon Kids Institute, The University of Western Australia, Crawley, Australia; Queensland Children’s Medical Research Institute, The University of Queensland, Brisbane, Australia

**Keywords:** Asthma, Atopy, House dust mite, Microarray, Raine study, CD4 T cell, Differential network analysis

## Abstract

**Background:**

Asthma is strongly associated with allergic sensitization, but the mechanisms that determine why only a subset of atopics develop asthma are not well understood. The aim of this study was to test the hypothesis that variations in allergen-driven CD4 T cell responses are associated with susceptibility to expression of asthma symptoms.

**Methods:**

The study population consisted of house dust mite (HDM) sensitized atopics with current asthma (*n* = 22), HDM-sensitized atopics without current asthma (*n* = 26), and HDM-nonsensitized controls (*n* = 24). Peripheral blood mononuclear cells from these groups were cultured in the presence or absence of HDM extract for 24 h. CD4 T cells were then isolated by immunomagnetic separation, and gene expression patterns were profiled on microarrays.

**Results:**

Differential network analysis of HDM-induced CD4 T cell responses in sensitized atopics with or without asthma unveiled a cohort of asthma-associated genes that escaped detection by more conventional data analysis techniques. These asthma-associated genes were enriched for targets of STAT6 signaling, and they were nested within a larger coexpression module comprising 406 genes. Upstream regulator analysis suggested that this module was driven primarily by IL-2, IL-4, and TNF signaling; reconstruction of the wiring diagram of the module revealed a series of hub genes involved in inflammation (IL-1B, NFkB, STAT1, STAT3), apoptosis (BCL2, MYC), and regulatory T cells (IL-2Ra, FoxP3). Finally, we identified several negative regulators of asthmatic CD4 T cell responses to allergens (e.g. IL-10, type I interferons, microRNAs, drugs, metabolites), and these represent logical candidates for therapeutic intervention.

**Conclusion:**

Differential network analysis of allergen-induced CD4 T cell responses can unmask covert disease-associated genes and pin point novel therapeutic targets.

**Electronic supplementary material:**

The online version of this article (doi:10.1186/s12920-016-0171-z) contains supplementary material, which is available to authorized users.

## Background

Asthma is a complex and heterogeneous disease that is characterized by airways inflammation, airways remodeling, and reversible airflow obstruction. The most common form of the disease begins in early childhood, and is associated with the development of sensitization to inhalant allergens such as house dust mites, pollen, and fungal spores [1]. The airways of atopic individuals with asthma are characterized by infiltration of mast cells, CD4 T cells and eosinophils, which are activated by exposure to allergens. CD4 T cells play a central role in the disease process by producing Th2 effector cytokines (e.g. IL-4, IL-5, IL-9, IL-13) that drive many of the hallmark phenotypic changes observed in asthma; these include airways hyperresponsiveness, increased mucus production, mucus cell hyperplasia, and eosinophilic inflammation. CD4 T cell responses are themselves heterogeneous, comprising multiple subsets that can either promote (Th1, Th2, Th9, Th17) or negate (Treg, Tr1) the airway inflammatory processes that underpin asthma [2–5].

There is compelling evidence supporting a causal relationship between IgE and Th2 cytokines and the expression of asthma symptoms [6–8]. However, the vast majority of atopics do not develop asthma, and the mechanisms that determine why some atopics develop asthma whilst others do not are not well understood. We have previously investigated this question via immunological profiling studies in a community cohort, and our findings showed that expression of asthma symptoms amongst atopics sensitized to HDM was associated with increased levels of HDM-specific IgE, blood eosinophils, and HDM-driven Th2 cytokine expression [9]. It is noteworthy that these previous studies were based on assessment of a restricted number of immunological parameters. In the present study we conducted a genome-wide analysis of HDM-driven CD4 T cell responses in sensitized atopics who were stratified on the basis of current asthma symptom expression. Our findings demonstrate that differential network analysis can unmask asthma-associated genes that escape detection by more conventional analytical approaches. Moreover, we illustrate the application of causal analytical algorithms to identify molecular drivers of the gene expression patterns, and pin point logical candidates for therapeutic intervention [10].

## Methods

### Study population

The study design was based on case/control comparisons of 72 subjects nested within the 14 year follow-up of The Western Australian Pregnancy (Raine Study) Cohort, an unselected longitudinal birth cohort, representative of the West Australian population [11]. The study population was stratified into three groups; HDM-sensitized atopics with current asthma (*n* = 22), HDM-sensitized atopics without current asthma (*n* = 26) and HDM-nonsensitized controls (without current asthma, *n* = 24). Current asthma was assessed by questionnaire, and was defined as a doctor diagnosis of asthma ever, plus the use of any asthma medication in the last 12 months, plus wheeze in the last 12 months. Total IgE was measured by ImmunoCAP (Phadia) from serum samples for all participants, as was Phaditop IgE; the Phadiatop test (Phadia) uses an ImmunoCAP with a balanced mix of representative inhalant allergens. Specific IgE to the following allergens was measured (ImmunoCAP, Phadia): HDM (*Dermatophagoides pteronyssinus*), rye grass pollen (*Lolium perenne*), cat, couch grass (*Cynodon dactylon*), mold mix (*Penicillium notatum, Cladosporium herbarum, Aspergillus fumigatus, Candida albicans, Alternaria alternata, and Helminthosporium halodes*), peanut, and food mix (egg white, milk, fish, wheat, peanut, and soybean). Sensitization to an allergen was defined as specific IgE ≥0.35kU/L.

### Cellular immunology

Cryobanked peripheral blood mononuclear cells (PBMC) were thawed and cultured in AIM-V medium (Gibco) in the presence or absence of 10 μg/ml HDM extract (CSL, Australia) as described previously [9, 12]. At the termination of the 24 h cultures, CD8 positive cells were removed and CD4 positive T cells were purified via positive immunomagnetic selection (Dynal Biotech) [13, 14]. The purity of the isolated CD4 T cells was routinely 98 %.

### Gene expression profiling

Total RNA was extracted from CD4 T cells using TRIzol (Invitrogen) followed by purification on an RNeasy column (Qiagen) [13, 14]. The integrity of the RNA was assessed on the Bioanalyzer (Agilent) and the RNA integrity number was greater than 8.0 for all samples. Total RNA (100 ng) was labelled and hybridized to Human Gene 1.0 ST microarrays (Affymetrix), employing standardized reagents and protocols from Affymetrix. The raw microarray data are available from the ncbi gene expression omnibus repository (GSE73482; http://www.ncbi.nlm.nih.gov/geo/).

### Data analysis

The raw microarray data was preprocessed in R employing the robust multi-array average (RMA) algorithm [15]. A custom mapping of probe sets to genes was utilized to annotate the arrays using current genome knowledge (hugene10sthsentrezgcdf, version 19, http://brainarray.mbni.med.umich.edu/) [16]. The quality of the microarray data was assessed with ArrayQualityMetrics, and low quality samples were removed from the analysis [17]. Batch effects were identified using principal components analysis. Two sample batches were identified, that were highly correlated with the microarray hybridization date and removed using the ComBat algorithm [18]. Noisy probe sets were identified using the proportion of variation accounted for by the first principal component (PVAC) algorithm (threshold = 0.4) and removed from the analysis [19]. The PVAC filtering step was applied separately to each of the three clinical groups, resulting in the identification of 1442 probe sets that were high quality in at least one clinical group, which were retained in the analysis. It is noteworthy that this filtering step results in a dramatic reduction of the number of probe sets remaining in the analysis (the unfiltered data contains 19,700 probes sets). This highly stringent filtering step is motivated by statistical modelling of Affymetrix probe level data that has demonstrated that the vast majority of probes do not measure any signal above background [20, 21]. The PVAC algorithm identifies unreliable/noisy probe sets as those in which the signal is not consistent across the multiple independent probes within a probe set [19].

Differentially expressed genes were identified using linear models for microarray analysis (LIMMA), with False Discovery Rate (FDR) adjustment for multiple testing [22]. Paired comparisons were performed to identify HDM regulated genes within each clinical group. Response patterns between clinical groups were based on unpaired comparisons of gene expression log ratios (HDM stimulated CD4 T cells/unstimulated controls).

The weighted gene coexpression network analysis (WGCNA) algorithm was used to construct a coexpression network [14]. Separate coexpression networks were constructed for each clinical group, using the same set of input genes (all 1442 probe sets identified by PVAC). Thus the resulting networks will be comprised of both constitutively expressed genes and differentially expressed genes. The standard WGCNA algorithm employs an absolute measure of the pairwise correlations between genes across the samples to build networks, and therefore no distinction is made between positive or negative correlations. In this study we used the signed variant of the WGCNA algorithm, which employs a transformation of the correlations that retains positive correlations and pushes negative correlations towards zero [23]. The signed correlation similarity between a pair of genes I and j across the samples (s_ij_) is defined as: ${s}_{jj}^{\kern1em  signed}=1+\frac{\mathrm{cor}\left(\mathrm{xi},\ \mathrm{x}\mathrm{j}\right)}{2}$

Modules of coexpression genes identified by WGCNA were tested for enrichment of differentially expressed genes by plotting the –Log10 p-values derived from the LIMMA analysis on a module-by-module basis. The wiring diagram of disease-associated modules was reconstructed employing prior knowledge from the Ingenuity Systems Knowledgebase [10, 24].

Differential network analysis was based on the methodology developed by Fuller et al. [25]. The approach entails case/control comparisons of gene expression patterns along dimensions of differential expression versus differential coexpression. Differential expression is calculated between cases and controls using a standard *t*-test. Differential coexpression is derived from comparing gene network connectivity measures between case/control networks. The network connectivity is defined as the sum of the signed correlation similarity of each gene with all other genes. These connectivity values are calculated separately for each clinical group, scaled by their maximum value to facilitate comparisons between groups, and then subtracted to define the difference. The differential expression/coexpression values are plotted as a scatterplot, and the plot is divided into 8 significance regions, defined by *t*-test statistic >1.96 (or less than – 1.96) and/or differential connectivity >0.3 (or less than -0.3). The number of observations that fall within each region is counted, and the statistical significance is assessed by comparing these values with the same values generated from 1000 random permutations of the sample class labels (i.e. case/control status).

Molecular drivers of the gene expression responses were identified utilizing upstream regulator analysis (Ingenuity Systems) [10]. Gene lists were analysed for enrichment of transcription factor target genes employing Enrichr [26]. Biological pathways enriched in the gene lists and modules were interrogated using cluster profiler to analyse the reactome pathway database [27].

## Results

### Study population

This study examined participants in the age 14 year Raine Study follow-up comprising three distinct phenotypic groups: HDM-sensitized atopics with current asthma (*n* = 22), HDM-sensitized atopics without current asthma (*n* = 26) and HDM-nonsensitized controls (without current asthma, *n* = 24). The characteristics of these participants are presented in Table 1. Although total IgE titre was significantly higher in the two HDM-sensitized groups than in the HDM-nonsensitized control group, it did not differ significantly between the two HDM-sensitized groups; HDM-specific IgE titre followed the same pattern, as did the number of allergens to which participants were sensitized. In contrast, Phadiatop IgE levels differed significantly between all three groups and were highest among asthmatics and lowest among HDM-nonsensitized controls. HDM-sensitized asthmatics had a higher prevalence of positive family history of atopy and of current rhinoconjunctivitis than the other two groups. There were, however, no differences in age, gender, height, weight, BMI, current smoking, or FEV1/FVC ratio between the three study groups.

**Table 1:** Characteristics of the study population

	HDM-nonsensitized controls	HDM-sensitized without asthma	HDM-sensitized with asthma	*P*-value
Number of participants	24	26	22	
Male (%)	50.0	42.3	63.6	0.333
Age at assessment (years)	14.1 (0.2)	14.1 (0.2)	14.1 (0.1)	0.912
Wheeze in past 12 months [A] (%)	0.0 a	0.0 a	100 b	N/A
Doctor diagnosis of asthma ever [B] (%)	29.2 a	26.9 a	100.0 b	<0.001
Asthma medication use in past 12 months [C] (%)	0.0 a	19.2 a	100.0 b	<0.001
Current medicated asthma [Postive for A,B &C] (%)	0.0 a	0.0 a	100.0 b	<0.001
Bronchial hyperresponsiveness (%)	17.4 a	28.0 a,b	59.1 b	0.009
Total IgE (kU/L)	21.2 a (23.3)	192.7 b (266.3)	479 b (629.7)	<0.001
Phadiatop IgE (Phadiatop units)	0.0 a (0.9)	21.4 b (30.6)	74.9 c (127.0)	<0.001
Number of allergens to which sensitized	0.0 a (1.7)	2.5 b (3.0)	3.0 b (4.0)	<0.001
House dust mite IgE (kU/L)	0.0 a (0.0)	27.1 b (33.0)	84.1 b (160.0)	<0.001
House dust mite sensitized^a^ (%)	0.0 a	100.0 b	100.0 b	<0.001
Rye grass sensitized (%)	20.8 a	53.8 b	63.6 b	0.009
Rye grass IgE (kU/L)	0.0 (0.2)	0.4 (4.6)	0.8 (6.0)	0.037*
Cat sensitized (%)	4.2 a	34.6 b	40.9 b	0.009
Cat IgE (kU/L)	0.0 a (0.0)	0.0 b (0.7)	0.3 b (2.9)	<0.001
Couch grass sensitized (%)	20.8	38.5	54.5	0.061
Couch grassIgE (kU/L)	0.0 (0.1)	0.1 (1.2)	0.5 (1.9)	0.056
Mould sensitized (%)	16.7	19.2	18.2	0.972
Mould mix IgE (kU/L)	0.0 (0.0)	0.0 (0.1)	0.0 (0.20)	0.293
Peanut sensitized (%)	0.0 a	11.5 a,b	31.8 b	0.007
Peanut IgE (kU/L)	0.0 a (0.0)	0.0 a,b (0.2)	0.1 b (0.5)	0.001
Food mix sensitized (%)	0.0	11.5	18.2	0.107
Food mix IgE (kU/L)	0.0 a (0.0)	0.1 b (0.2)	0.2 b (0.2)	<0.001
Positive family history of atopy (%)	61.9 a	63.6 a	95.5 b	0.018
FEV/FVC_1_ (% ratio)	91.8 (8.6)	92.1 (10.9)	91.9 (17.8)	0.813
Current rhinoconjunctivitis (%)	16.7 a	0.0 a	68.2 b	<0.001
Maternal smoking in pregnancy (%)	22.2	31.6	25.0	0.801
Current smokers (%)	0.0	16.0	9.1	0.131
BMI	18.8 (4.3)	20.6 (4.9)	21.0 (4.2)	0.106
Height (cm)	166.8 (9.2)	166.0 (11.1)	161.6 (14.1)	0.246
Weight (kg)	50.5 (17.8)	57.1 (19.8)	54.3 (15.2)	0.278

Median (interquartile range) is displayed for all continuous measures. *P* value is derived from analyses comparing the three groups: prevalence values were compared by Chi square analysis; continuous measures were compared by Kruskal Wallis analysis. Where significant differences were observed between the three group groups (*P* < 0.05 in table) subsequent pairwise comparisons were performed; identical letters denote groups that do not differ significantly at the 0.05 level after adjusting for multiple comparisons (a vs a = not different; a vs b = significantly different). ^a^Sensitization was defined as specific IgE ≥0.35 kU/L). *Kruskal Wallis analysis found that the three groups were not the same but subsequent pairwise comparisons did not show significant differences (*P* < 0.05) between groups after P was adjusted for multiple testing

### Molecular profiling of allergen-driven gene expression profiles in CD4 T cells

PBMC from the subjects in Table 1 were cultured in the presence or absence of HDM allergen extracts for 24 h, after which, CD4 T cells were isolated by immunomagnetic separation, and gene expression patterns were profiled on microarrays, as per our earlier studies [13, 14]. The microarray data was preprocessed and summarized into a set of non-redundant genes, and stringent filtering was employed to filter out noisy probe sets, resulting in 1442 remaining genes for analysis (see Methods).

**Fig. 1:**
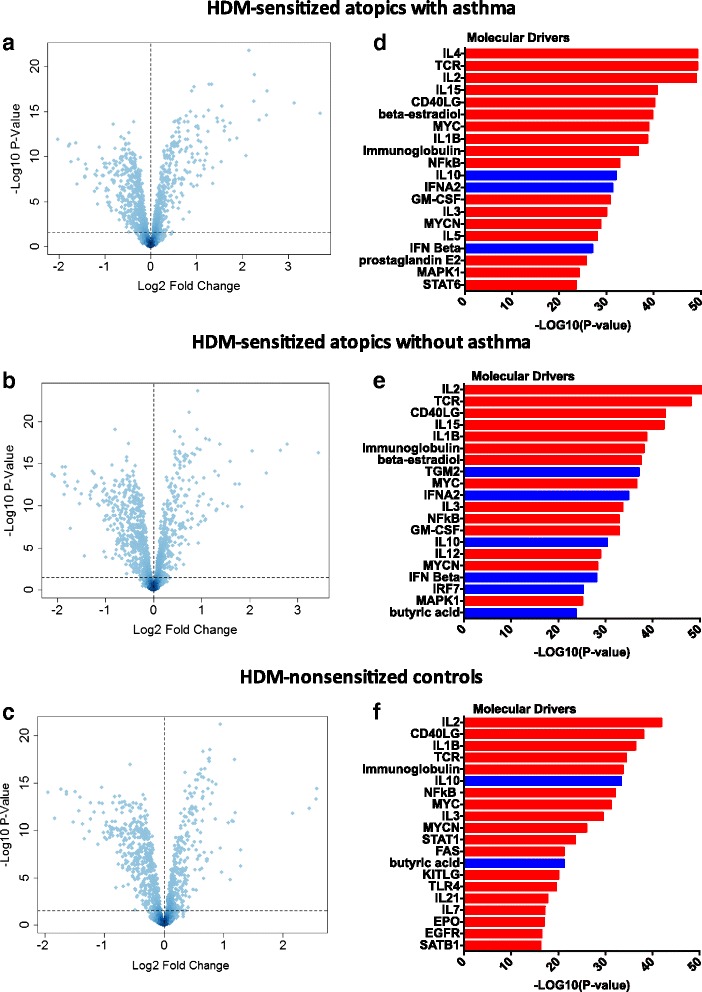
Identification of molecular drivers of HDM-driven gene expression patterns in CD4 T cells. Differentially expressed genes/molecular drivers were identified in: **a**/**d** HDM-sensitized atopics with asthma; **b**/**e** HDM-sensitized atopics without asthma; and **c**/**f** HDM-nonsensitized controls. Data analysis was performed by LIMMA (**a**, **b**, **c**) and upstream regulator analysis (**d**, **e**, **f**). The dashed horizontal line in figs **a**, **b**, **c** indicates FDR <0.05. Drivers in red are predicted to be activated and those in blue are inhibited

First, we compared gene expression patterns between HDM-stimulated and unstimulated CD4 T cells from HDM-sensitized asthmatics. The data showed that 409 genes were upregulated and 452 genes were downregulated after adjustment for multiple testing (FDR <0.05, Fig. 1a). The 50 most statistically significant differentially expressed genes (DEG) are shown in Additional file 1: Table S1, with IL4R as the top ranked DEG. Next, we compared HDM-stimulated and unstimulated CD4 T cells in HDM-sensitized subjects without asthma. The data showed that 407 genes were increased and 503 genes were decreased (FDR <0.05, Fig. 1b). The 50 most significant DEG are shown in Additional file 2: Table S2, with IL4R absent as it is ranked number 85. Finally, we examined the response to HDM in nonsensitized controls, and we found that 348 genes were upregulated and 475 were downregulated (FDR <0.05, Fig. 1c). Additional file 3: Table S3 lists the 50 most statistically significant DEG and a change in gene expression of IL4R was not detected. It is noteworthy that there was considerable overlap in the respective responses when comparing stimulated versus unstimulated CD4 T cells for each group (Additional file 4: Figure S1). Moreover, this overlap extended to the level of biological pathways (Additional file 5: Figure S2).

### Molecular drivers of allergen-driven gene expression profiles in CD4 T cells

We employed upstream regulator analysis to identify the putative molecular drivers of the gene expression patterns in CD4 T cells [10]. This analysis leverages experimental findings from prior studies to identify the putative regulatory molecules that are driving the observed differential expression patterns. Two statistical measures are calculated: (i) the overlap p-value measures enrichment of target genes amongst the list of differentially expressed genes (both upregulated and downregulated); (ii) the activation Z-score measures the pattern match between the observed gene expression changes (up/down regulation) and the predicted pattern based on prior knowledge. (an activation Z-score >2 is statistically significant) [10]. As illustrated in Fig. 1d, the most significant candidate drivers of the responses in HDM-sensitized asthmatics were IL-4, TcR signaling, IL-2, IL-15, CD40L, and IL-1B. This analysis also suggested that IL-10 and type I interferon signaling was downregulated by HDM stimulation. A similar pattern was observed in the responses from HDM-sensitized atopics without asthma (Fig. 1e), although the activation Z-score for IL-4 did not reach statistical significance (Z-score = 1.79). In the HDM-nonsensitized controls, the putative molecular drivers of the HDM responses were IL-2, CD40L, IL-1B, and TcR signaling (Fig. 1f). IL-10 and butyric acid were identified as candidate negative regulators of this response.

HDM extracts contain non-allergenic components such as LPS that can stimulate immune responses [28, 29]. In this context it is noteworthy that LPS was not identified as a significant candidate driver of the responses in any of the three groups (activation Z-score ~ 1.0).

### Differential analysis of HDM-driven CD4 T cell responses in sensitized atopics with or without asthma and in non-sensitized controls

**Fig. 2:**
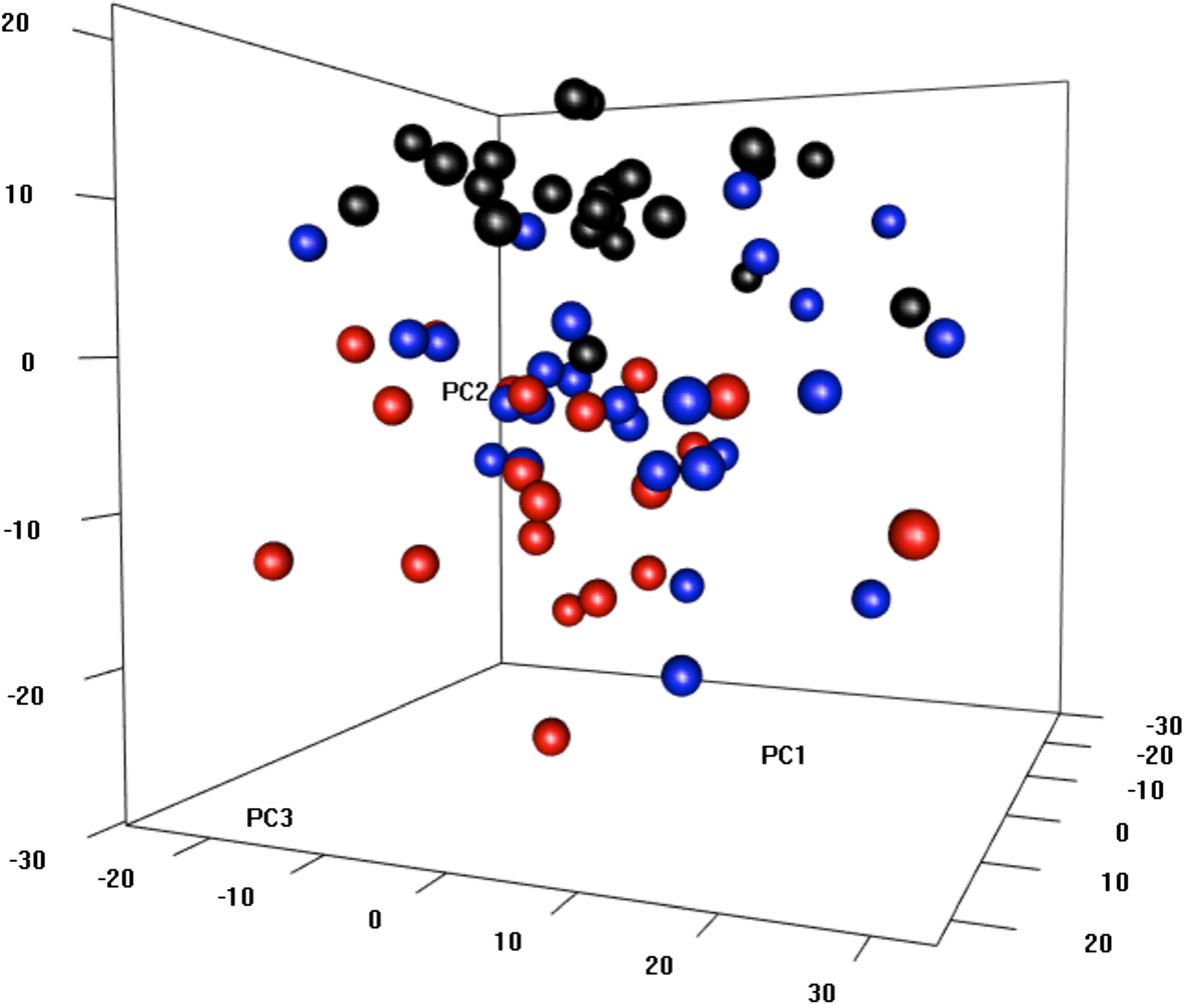
HDM response patterns in CD4 T cells from sensitized atopics and nonsensitized controls. Gene expression log ratios were calculated for each subject (HDM stimulated CD4 T cells/unstimulated CD4 T cells) and analyzed by principal components analysis. HDM-sensitized atopics with asthma are shown in red, HDM-sensitized atopics without asthma are blue and HDM-nonsensitized controls in black circles

To provide a global view of the HDM responses, we calculated gene expression log ratios (HDM stimulated T cells/unstimulated T cells) for each subject, and analyzed the data using principal component analysis. As illustrated in Fig. 2, the data showed that the HDM-sensitized atopics with asthma (red circles) clustered separately to the HDM-nonsensitized controls (black circles). In contrast, HDM-sensitized atopics without asthma did not form an isolated cluster, but instead blended into the two other groups.

**Fig. 3:**
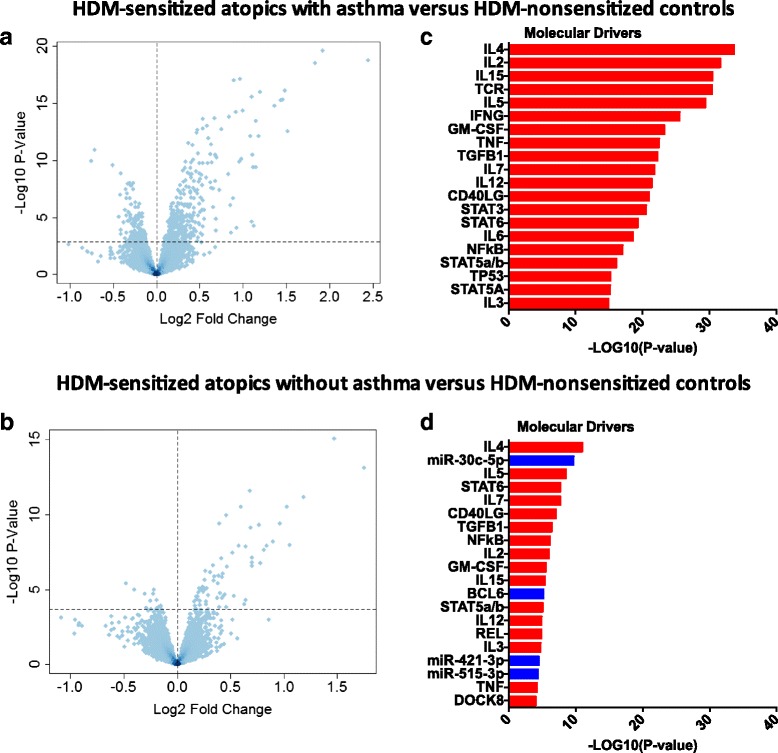
Identification of molecular drivers of differential responses to HDM in CD4 T cells. Differentially expressed genes/molecular drivers were identified between the response patterns of: **a**/**c** HDM-sensitized atopics with asthma versus HDM-nonsensitized controls; **b**/**d** HDM-sensitized atopics without asthma versus HDM-nonsensitized controls. Data analysis was performed by LIMMA (**a**, **b**) and upstream regulator analysis (**c**, **d**). The dashed horizontal line in figs **a**, **b** indicates FDR <0.05. Drivers in *red* are predicted to be activated and those in *blue* are inhibited

We then proceeded to identify differentially expressed genes between the responses of the three groups. This was done by comparing gene expression changes in HDM-stimulated CD4 T cells between groups. Reflecting the findings from the PCA analysis; 535 genes were differentially expressed between HDM-sensitized atopics with asthma compared to the HDM-nonsensitized controls (Fig. 3a); less genes (79 genes) were differentially expressed between HDM-sensitized atopics without asthma and the nonsensitized controls (Fig. 3b); and no genes were differentially expressed between HDM-sensitized atopics with or without asthma (data not shown). Although this last comparison did not reveal any differences, the molecular drivers underlying the differential responses of the two HDM-sensitized groups versus the nonsensitized controls were not the same (Fig. 3c, d). For instance, the asthmatic responses included highly ranked proinflammatory pathways such as IFNg, TNF, STAT3, and IL-6 (Fig. 3c). In contrast, the molecular drivers underlying the differential response to HDM-sensitized nonasthmatics versus nonsensitized controls were much less significant and/or mainly restricted to Th2-assocaited signaling pathways (IL-2, IL-4, IL-5, STAT6, Fig. 3d).

### Coexpression network analysis of HDM-driven CD4 T cell responses

**Fig. 4:**
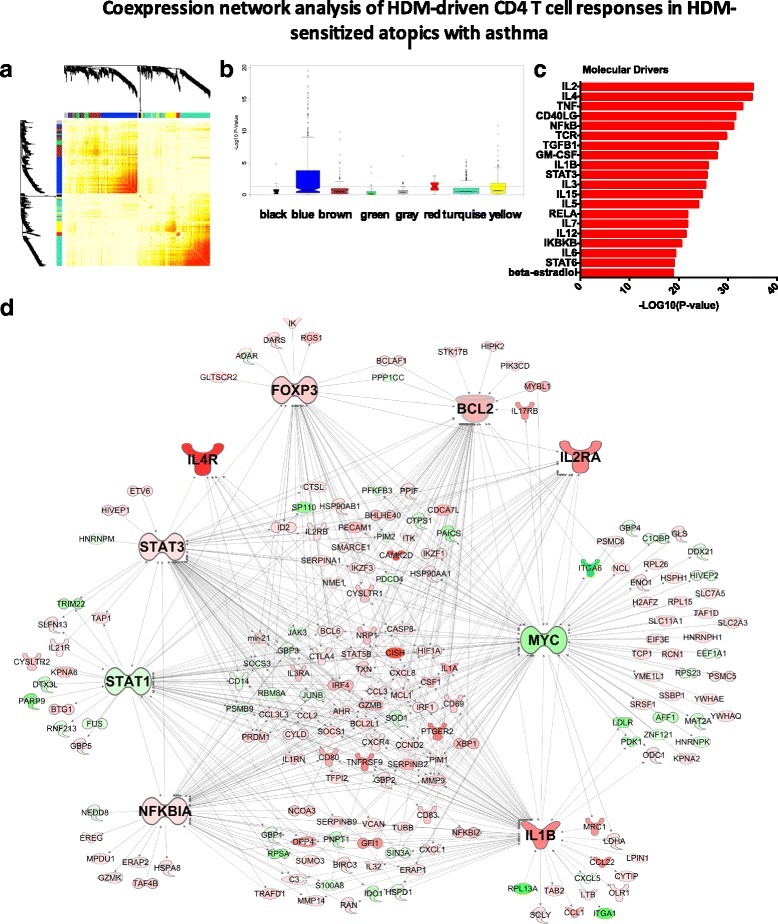
Coexpression networks underling HDM-driven CD4 T cell responses in sensitized asthmatics. **a** Heatmap illustrating the gene coexpression network. Increasing red intensity indicates increasing strength of correlation between genes. Modules are defined as the branch-like structures on the dendrogram. **b** Differentially expressed genes were identified between HDM-driven CD4 T cell responses in sensitized asthmatics versus nonsensitized controls and the –Log10 p-values were plotted on a module by module basis. **c** Molecular drivers of the blue modules were identified employing upstream regulator analysis. **d** The wiring diagram of the blue module was reconstructed employing prior knowledge from the Ingenuity Systems database

To explore the CD4 T cell response to HDM at the systems-level, we employed coexpression network analysis [14, 24]. We constructed the coexpression network underlying the HDM-stimulated CD4 T cell response in the HDM-sensitized asthmatics. The resulting network was organized into 7 coexpression modules (Fig. 4a), which were functionally distinct (Additional file 6: Figure S3) To identify disease-associated modules, gene expression levels were compared between the HDM-sensitized asthmatics versus the nonsensitized controls, and the –log10 P-values derived from this analysis were plotted on a module-by-module basis (Fig. 4b). The data showed that one module in particular (the “blue module”) was enriched with differentially expressed genes. This module contained 406 genes, and upstream regulator analysis suggested the underlying molecular drivers were IL2, IL4, TNF, CD40LG and NFkB (Fig. 4c). To obtain additional information about the regulation and function of this module, we employed experimental findings from published studies to reconstruct the wiring diagram of the module [24]. This analysis unveiled a series of hub genes including MYC (96 connections to other genes; i.e. edges), IL-1B (85 edges), STAT3 (76 edges), STAT1 (58 edges) NFkBIA (61 edges), BCL2 (55 edges) FOXP3 (41 edges), IL-2Ra (22 edges) and IL-4R (8 edges) (Fig. 4d).

**Fig. 5:**
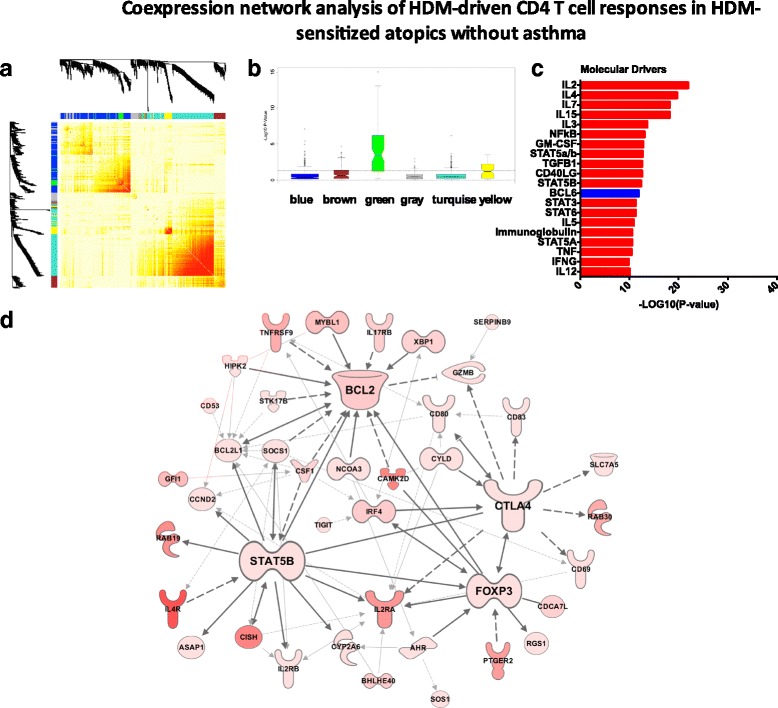
Coexpression networks underling HDM-driven CD4 T cell responses in sensitized atopics without asthma. **a** Heatmap illustrating the gene coexpression network. Increasing red intensity indicates increasing strength of correlation between genes. Modules are defined as the branch-like structures on the dendrogram. **b** Differentially expressed genes were identified between HDM-driven CD4 T cell responses in sensitized atopics without asthma versus nonsensitized controls and the –Log10 p-values were plotted on a module by module basis. **c** Molecular drivers of the green modules were identified employing upstream regulator analysis. **d** The wiring diagram of the green module was reconstructed employing prior knowledge from the Ingenuity Systems database

In parallel, we constructed the coexpression network underlying CD4 T cell responses in HDM-sensitized atopics without asthma (Fig. 5a). Five functional modules were identified (Additional file 7: Figure S4), and one of these modules (the “green” module) was differentially expressed between HDM-sensitized atopics without asthma and the nonsensitized controls (Fig. 5b). This module contained 85 genes, 95 % of which were also identified in the asthmatic blue module described above. The putative molecular drivers of this module were IL-2, IL-4, and IL-7, IL-15, and IL-3 (Fig. 5c). Reconstruction of the module using prior knowledge unveiled the hub genes STAT5B (14 edges), BCL2 (14 edges), CTLA4 (11 edges) and FOXP3 (10 edges, Fig. 5d).

### Differential network analysis of HDM-driven CD4 T cell response patterns in sensitized atopics with or without asthma

The above analyses suggested that HDM-driven gene network patterns were not equivalent in HDM-sensitized atopics who did or did not have asthma. However these analyses were based on parallel comparisons of the two HDM-sensitized cohorts with the nonsensitized control group, rather than a direct comparison between the two HDM-sensitized cohorts. To address this issue, we employed differential network analysis to directly compare the two groups [25]. The approach entails case/control comparisons of gene expression patterns along axes of differential expression versus differential coexpression (see Methods). The resulting plot is divided into eight regions; genes that appear in regions R2 and R6 are differentially expressed (but not differentially coexpressed); genes in regions R4 and R8 are differentially coexpressed (but not differentially expressed), and genes in the remaining regions (R1, R3, R5, R7) are both differentially expressed and differentially coexpressed. The number of observations that fall within each region is counted, and statistical significance is assessed by a simulation which randomly permutes or changes the case/control status of the subjects and runs the analysis. This simulation is repeated 1000 times and region counts are then compared between the original observed data and the randomized/permutated data.

**Fig. 6:**
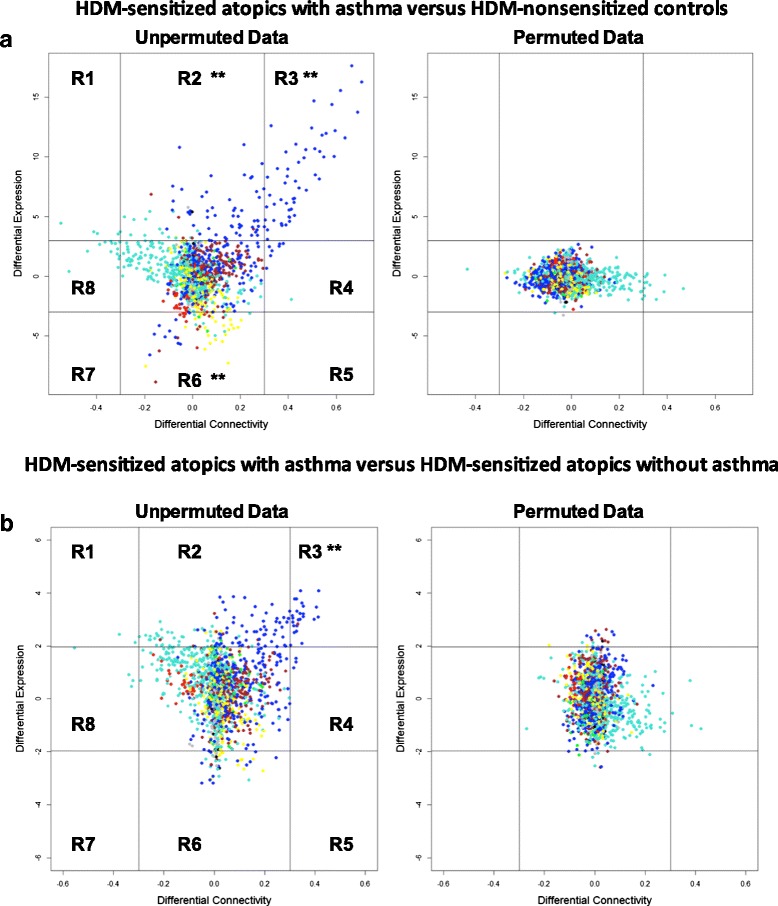
Differential network analysis of CD4 T cell responses to HDM in sensitized atopics with asthma versus HDM-nonsensitized controls. Gene expression patterns were compared along dimensions of differential expression versus differential connectivity; **a** HDM-sensitized atopics with asthma versus HDM-nonsensitized controls; **b** HDM-sensitized atopics with asthma versus HDM-sensitized atopics without asthma. To assign statistical significance to the unpermuted data, we permute the case/control status of the data 1000 times. Region counts can then be compared between permuted and unpermuted data. The data points are colored by their module assignment. Genes in the region 3 of the plots have upregulated expression levels and connectivity patterns in the subjects with asthma. ** *P* < 0.005

First, we employed this approach for differential network analysis of the responses in HDM-sensitized atopics with asthma versus HDM-nonsensitized controls. The data showed that the genes with upregulated expression and coexpression (region 3, *P* = 0.0009) patterns in the asthmatic group were exclusively from the blue module (Fig. 6a, Additional file 8: Table S4). Then we employed differential network analysis to compare the responses in HDM-sensitized atopics with or without asthma. The data showed that although the differences were more subtle, 25 genes were associated with asthma (region 3, *P* = 0.0009, Fig. 6b, Additional file 9: Table S5). Notably, all 25 of these genes were upregulated in the responses of both HDM sensitized cohorts, and 14 out of 25 were also upregulated in the responses of the HDM-nonsensitized controls, albeit to a lesser extent (Additional file 10: Table S6). In addition, it is pertinent to note with the exception of one gene “PRPS1” (brown module), these asthma-associated genes were again restricted to the blue module. Bioinformatics analyses showed that these asthma-associated genes were enriched with targets of STAT6 signaling (Additional file 11: Table S7). Finally, two additional genes of interest were identified in region 1 of Fig. 6b. These genes were; (i) gamma-aminobutyric acid A receptor-associated protein (GABARAP), which is a ubiquitin-like modifier with a role in the intracellular transport of the GABA(A) receptor and the regulation of apoptosis and autophagy, and (ii) macrophage-expressed gene 1 (MPEG1), which encodes a perforin-like protein with bactericidal activity.

### Identification of negative regulators of HDM-induced CD4 T cell responses in sensitized atopics with asthma

**Fig. 7:**
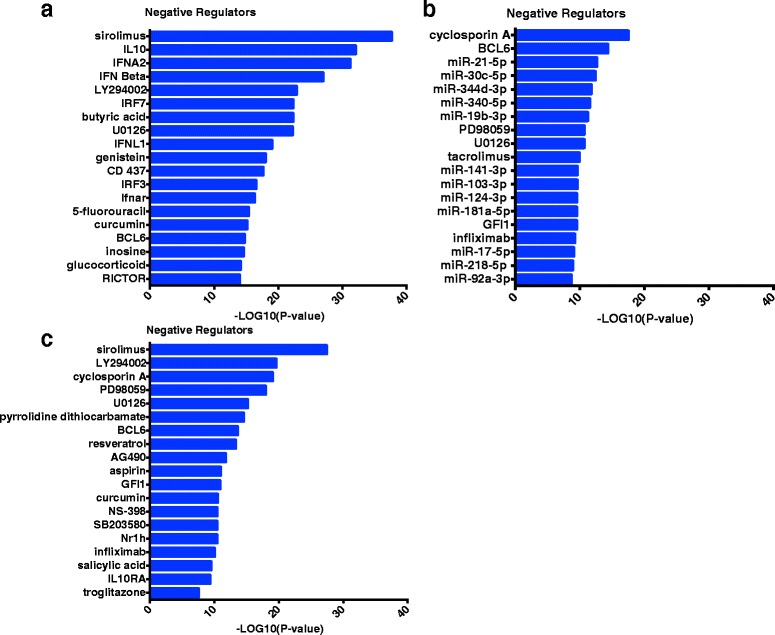
Identification of negative regulators of the house dust mite response in CD4 T cells from atopics with asthma. Negative regulators were identified in: **a** HDM-sensitized atopics with asthma **b** HDM-sensitized atopics with asthma versus HDM-nonsensitized controls; **c** The blue module in HDM-sensitized atopics with asthma. Upstream regulator analysis was used

Finally, we employed upstream regulator analysis to identify negative regulators of HDM-driven CD4 T cell responses in sensitized atopics with asthma. Three separate comparisons were performed; (i) HDM-stimulated versus unstimulated CD4 T cells within the HDM-sensitized asthmatics (Fig. 7a); (ii) HDM responses of HDM-sensitized asthmatics versus HDM-nonsensitized controls Fig. 7b); (iii) the blue asthma-associated coexpression module identified in Fig. 4 using coexpression network analysis (Fig. 7c). This analysis highlighted several molecular pathways (IL-10, type I interferon, microRNAs), drugs (sirolimus, LY294002, glucocorticoids), and metabolites (butyric acid, curcumin), which have potential utility as targets for therapeutic intervention.

## Discussion

Atopic asthma is thought to be driven to a significant extent by repeated cycles of allergen-driven, Th2-associated inflammation. However, it is not known why the vast majority of atopics who are sensitized to ubiquitous environmental allergens do not develop persistent asthma. In this regard we hypothesized that variations in allergen-driven CD4 T cell responses may in part determine expression of disease symptoms. Our findings showed that HDM responses from sensitized atopics with or without asthma were not different when analyzed with conventional statistical methods that focus solely on differences in gene expression levels. In contrast, interrogation of the same data using differential network analysis, unmasked a cohort of asthma-associated genes. These asthma genes were enriched for targets of STAT6 signaling and they were nested within a larger coexpression module comprising 406 genes. Upstream regulator analysis suggested that this module was driven primarily by IL-2, IL-4 and TNF signaling. The module was also characterized by the presence of a series of hub genes, which were involved in the regulation of inflammation (IL-1B, NFkB, STAT1, STAT3), apoptosis (Bcl2, Myc), and regulatory T cell function (IL-2Ra, FoxP3). Finally, we identified several negative regulators of asthmatic CD4 T cell responses to allergens (e.g. IL-10, type I interferons, microRNAs, drugs, metabolites), and these represent logical candidates for therapeutic intervention.

The asthma-associated genes we unveiled by differential network analysis shown in Additional file 9: Table S5 and Additional file 10: Table S6, play diverse roles in biology, but many of them have unknown roles in allergy and asthma: (i) CNKSR2 functions as a scaffold that regulates mitogen-activated protein kinase signaling and deficiency in CNKSR2 is characterized by intellectual disability and seizures, [30] (ii) DACT1 is known to regulate Wnt/beta-catenin signaling, a pathway that is essential for normal development [31] and for gata-3 expression and Th2 differentiation; [32] DACT1 may therefore regulate Th2 differentiation, (iii) DARS forms a complex with other enzymes that mediate attachment of amino acids to tRNAs; mutations in DARS cause hypomyelination of cerebral white matter which manifests clinically as severe leg spasticity, [33] (iv) KPNA6 regulates the nuclear import of STAT1 and STAT3, which were hub genes in the asthma module, [34] (v) NCOA3 is a nuclear receptor coactivator that negatively regulates NFkB signaling and inflammatory responses, [35, 36] (vi) NDFIP2 activates HECT domain-containing E3 ubiquitin-protein ligases, and promotes IFNg production in Th1 cells, [37] (vii) NFkBIZ is a transcription factor that interacts with RORgammaT and RORalpha; it drives Th17 development, but is dispensable for Th1 and Th2 development, [38] (viii) PRPS1 is an enzyme that catalyzes the synthesis of phosphoribosylpyrophosphate, which is required for the synthesis of nucleotides; mutations in this gene are associated with multiple disorders including neuropathy, hearing loss, and central nervous system impairment [39] (ix) RASGRP3 is a guanine nucleotide exchange factor that activates Ras and Rap1 signaling; it negatively regulates TLR-induced IL-6 production in macrophages, [40] (x) TRAFD1 is an interferon-induced gene that negatively regulates innate immune responses to LPS and Poly-IC [41]. Detailed mechanistic studies will need to be conducted on all these genes to define their role in allergy and asthma.

To identify molecular drivers (and negative regulators) of CD4 T cell responses to allergens, we considered three separate analyses: (i) HDM-stimulated versus unstimulated CD4 T cell responses within HDM-sensitized asthmatics (Fig. 1d); (ii) the differential response to HDM in sensitized asthmatics versus non-sensitized controls (Fig. 3c); (iii) and the asthma-associated module (Fig. 4c). In all three comparisons, IL-2 and IL-4 were the most significant drivers we identified. We have previously shown that dual inhibition of IL-2 and IL-4 signaling with neutralizing antibodies could silence allergen-driven Th2 responses in PBMC from sensitized atopics, thus confirming the findings from upstream regulator analysis [14]. Moreover, we showed that IL-2 drives expression of both Th2 cytokines and regulatory T cell (Treg) signature genes (FoxP3), suggesting that IL-2 may play a dual effector/regulatory role in allergic responses [14]. Notably, IL-2-mediated upregulation of IL-4Ra expression is required to prime CD4 T cells for Th2 differentiation and enhance Th2 cytokine expression [42]. IL-2 is also required for the generation and homeostasis of Tregs, and the maintenance of FoxP3 expression [43, 44]. In a clinical trial, blockade of IL-2Ra with a monoclonal antibody improved pulmonary function and asthma control [45]. IL-4/IL-4R antagonists have also been evaluated in clinical trials, but improvements in asthma symptoms were limited or inconsistent across studies [46, 47]. Our findings suggest that dual inhibition of IL-2 and IL-4 signaling may silence Th2 responses, but the potential benefits of this approach will need to be weighed up against the potential impact on Tregs.

Our data identified IL-15 as a key driver of the asthmatic responses (Figs. 1d, 3c and 4c). Like IL-2 and IL-4, IL-15 transduces signals via the common cytokine receptor gamma-chain (IL2RG, CD132). Mori et al. showed that exogenous IL-15 drives the expression of Th2 cytokines in HDM-specific CD4 T cell clones [48]. Ruckert et al. demonstrated that inhibition of IL-15 signaling with a soluble receptor antagonist abolished the induction of allergic airways inflammation, and reduced the production of allergen-specific IgE and airways hyper-reactivity [49]. IL-15 is therefore a logical therapeutic target for allergic asthma, however it is noteworthy that IL-15 may play a protective role during virus-induced asthma exacerbations [50, 51].

Upstream regulator analysis suggested that TNF was a highly significant molecular driver of the asthma-associated module (Fig. 4c). This finding is consistent with the paradigm proposed by Liu, who suggested that Th2 cells could be divided into two subsets; “inflammatory Th2 cells” that express Th2 cytokines plus TNF, versus “regulatory Th2 cells” that express Th2 cytokines plus IL-10 [52]. Our network analysis suggests that the concept of inflammatory Th2 responses may be extended to include additional proinflammatory pathways including IL-1B, STAT1, and STAT3 signaling (Fig. 4d). TNF blockade has been evaluated in clinical trials, but severe adverse events were reported including infection and malignancy [53]. IL-1B is an interesting therapeutic candidate because a broad range of acute and chronic inflammatory diseases respond to IL-1B blockade [54]. In IL-1R1 deficient mice, allergen-induced eosinophilic inflammation and goblet cell hyperplasia were reduced, highlighting the therapeutic potential of this pathway [55].

An alternative therapeutic strategy to antagonizing key drivers of the asthmatic responses is to induce negative regulators. Two plausible candidates for this approach, identified by our study, are type I interferons and IL-10 (Fig. 1d). Huber et al. showed that type I interferons inhibit Th2 development and cytokine secretion of committed Th2 cells in humans but not mice [56]. Type I interferons are an attractive therapeutic target because they may potentially protect asthmatics from virus-induced exacerbations [57]. IL-10 is a potent anti-inflammatory protein that is produced by regulatory T cells, and the generation of IL-10 producing Tregs during allergen-specific immunotherapy is thought to underpin the development of tolerance [58, 59]. Previous studies have shown that intranasal delivery of recombinant IL-10 protein or an IL-10 transgene can inhibit allergen-induced airways inflammation in mouse models [60, 61]. IL-10 has a short half-life in vivo, thus a practical cell based therapy could entail the generation of allergen-specific, IL-10 secreting Treg, via in vitro stimulation of CD4 T cells in the presence of glucocorticoids and 1α25-dihydroxyvitamin D3 [62, 63].

Another class of molecules we identified that has therapeutic potential are microRNAs (miRNAs, Fig. 7b), small endogenous RNAs that can regulate gene expression via mRNA degradation or translational repression [64]. Each miRNA can modulate hundreds of genes, and therefore even modest alterations to miRNA expression can have a dramatic impact on biological networks and functionality [64]. Mattes et al. showed that miR-126 is upregulated in the airways of a mouse model of HDM-driven allergic airways disease, and inhibition of miR-126 by an antagomir (cholesterol-linked, single-stranded antisense RNA) reduced HDM-induced allergic inflammation and abolished airways hyperreactivity [65]. Collison et al. showed that antagomir mediated silencing of miR-145 but not miR-21 or miR-let-7b inhibited HDM-induced eosinophilic inflammation, Th2 cytokine expression and airways hyperresponsiveness in a mouse model [66]. Moreover, they also showed that the effects of miR inhibition were comparable to dexamethasone treatment. Simpson et al. showed that miR-19-a expression was increased in CD4 T cells isolated from the bronchoalveolar lavage of human asthmatics. MiR-19a is transcribed as a polycistronic cluster of six miRNAs (miR-17/92 cluster). Th2 responses were impaired in CD4 T cells deficient for the miR-17/92 cluster, and this was restored by transfection of mature mimics of miR-19a or mi-19b [67]. Here we have identified a number of miRNAs that are predicted to inhibit differential responses to HDM in asthmatics versus non-sensitized controls (Fig. 7b). However as these findings are largely based on computational predictions from the TargetScan database, [68] mechanistic studies will be required to validate these data.

Additionally upstream regulator analysis identified curcumin, a natural phenol present in turmeric which has been shown to exhibit a variety of anti-inflammatory properties, as a potential negative regulator of the asthmatic response to HDM (Figs. 1d and 4c) [69]. Although curcumin is known for its low systemic bioavailability and is rapidly metabolized in the gut, intranasal delivery in a murine model of asthma reduced airways inflammation, histamine release, and eosinophil peroxidase activity in bronchoalveolar lavage fluid [70]. It has also been shown that daily administration of curcumin via intraperitoneal injection can attenuate allergen-induced airways inflammation and hyper-responsiveness [71].

We have also identified butyric acid, a short chain fatty acid (SCFA) produced by fermentation of dietary fiber by commensal gut microbiome, [72] as a potential negative regulator (Fig. 7a). SCFAs in the gut interact with G-protein coupled receptors (GPR), and Maslowski et al. has shown that SCFA-GPR43 signalling was required for the normal resolution of inflammatory responses in mouse models of colitis, asthma, and arthritis [73]. Additionally Trompette et al. demonstrated that mice fed a high fiber diet had higher levels of circulating SCFA (acetate, butyrate, propionate), and were protected from HDM-driven airways inflammation, and this effect could be reproduced by SCFA supplementation of drinking water [74]. Of interest in this context was the prominence here of butyrate in the list of negative regulators of the HDM response in both non-atopic controls and non-asthmatic HDM-sensitized atopics, in contrast to the HDM-sensitized asthmatics (Fig. 1e/f). This may imply that acquired or inherent resistance to the immunomodulatory effects of microbiome-derived SCFA may be one of the determinants of the pathogenic potential of Th2-associated gene networks underlying allergen-specific CD4 Th-memory responses amongst atopics.

This study has limitations that should be acknowledged. First, our studies focused on stimulating CD4 T cells in vitro outside of the context of the airways. This is a limitation because signals in the local microenvironment may modulate the CD4 T cell response. Moreover, our experimental strategy cannot provide any direct insight into the impact on these T cell responses on the target tissues in the airways. This limitation can be addressed by performing molecular profiling studies on CD4 T cells and epithelial cells isolated from airway biopsies after experimental allergen challenge [75]. Second, our analyses focused on a single time point (24 h) post HDM stimulation, and we have previously shown that later time points are optimal for detecting the expression of effector cytokines [13]. Third, network analysis requires larger sample sizes compared to conventional analyses that focus on differentially expressed genes, and therefore follow-up studies in a larger sample will have more power to find subtle differences. Finally, the differential network analyses we performed require group-wise comparisons, and this is a limitation because asthma is a heterogeneous disease that comprises multiple subphenotypes [3]. This limitation can be addressed by data analysis methods that can interpret genome-wide immune responses using data from a single subject [76]. Notwithstanding these limitations, our study illustrates the application of differential network analysis techniques to the unmasking of asthma-associated genes, and has highlighted novel candidate genes for functional dissection in mechanistic studies, and potential new opportunities for therapeutic intervention.

## Conclusion

Differential network analysis of immune profiling data unmasks asthma-associated genes that escape detection employing more conventional data analysis methods. Combining network analysis with upstream regulator analysis can predict the cause of the observed gene expression changes, and pin point novel therapeutic targets.

### Ethics

This study was approved by our institutional human ethics committee.

## Additional files

Additional file 1: Table S1. Fifty most significant differentially expressed genes in HDM-stimulated versus resting CD4 T cells from HDM-sensitized atopics with asthmatics. Gene expression patterns were compared between HDM-stimulated and unstimulated CD4 T cells from HDM-sensitized atopics with asthma. Here we present the 50 most significant differentially expressed genes. (XLS 34 kb) Additional file 2: Table S2. Fifty most significant differentially expressed genes in HDM-stimulated versus resting CD4 T cells from HDM-sensitized atopics without asthma. We compared gene expression changes in HDM-stimulated and unstimulated CD4 T cells from HDM-sensitized atopics without asthma. Here are the 50 most significant differentially expressed genes. (XLS 34 kb) Additional file 3: Table S3. Fifty most significant differentially expressed genes in HDM-stimulated versus resting CD4 T cells from HDM-nonsensitized controls. We compared gene expression changes in HDM-stimulated and unstimulated CD4 T cells from HDM-nonsensitized controls. The 50 most significant differentially expressed genes are shown here. (XLS 33 kb) Additional file 4: Figure S1. Overlap of differentially expressed genes by group. Overlap of differentially expressed genes in HDM-sensitized atopics with asthma (red), HDM-sensitized atopics without asthma (blue) and non-sensitized controls (black). Gene expression patterns in CD4^+^ T cells were profiled by microarray and we compared HDM stimulated versus unstimulated CD4^+^ T cells for each group. Numbers in red indicate upregulated genes and numbers in green represent downregulated genes. (PDF 68 kb) Additional file 5: Figure S2. Biological pathways enriched in the CD4 T cell responses to HDM from the three clinical groups. Data analysis by Cluster Profiler using reactome database. Biological pathways enriched in the set of upregulated (Upreg) and downregulated (Downreg) genes from HDM-stimulated versus resting CD4 T cells from HDM-sensitized atopics with/without asthma and HDM nonsensitized controls. (PDF 95 kb) Additional file 6: Figure S3. Biological pathways enriched in the WGCNA modules in the CD4 T cell responses to HDM from HDM sensitized atopics with asthma. Data analysis by Cluster Profiler using reactome database. (PDF 73 kb) Additional file 7: Figure S4. Biological pathways enriched in the WGCNA modules in the CD4 T cell responses to HDM from HDM sensitized atopics without asthma. Data analysis by Cluster Profiler using reactome database. (PDF 72 kb) Additional file 8: Table S4. Upregulated and differentially expressed genes in the HDM-sensitized atopics with asthma compared to HDM-nonsensitized controls. Differential network analysis of the responses in HDM-sensitized atopics with asthma versus HDM-nonsensitized controls identified genes with upregulated expression and coexpression (region 3, *P* = 0.0009) patterns in the asthmatic group. Genes identified were exclusively from the blue module (Fig. 6a). (XLS 32 kb) Additional file 9: Table S5. Asthma associated genes identified via differential network analysis of CD4 T cell responses to HDM. Genes with both upregulated expression and coexpression patterns (region 3, *P* = 0.0009) were identified in the HDM-sensitized atopics with asthma compared to HDM-sensitized atopics without asthma using differential network analysis. Genes identified were exclusively from the blue module (Fig. 6b) with the exception of *PRPS1 (brown module). (XLS 29 kb) Additional file 10: Table S6. Expression of asthma-associated genes in CD4 T cell responses to HDM in the three clinical groups. Asthma-associated genes were identified by differential network analysis (Fig. 6b). Log Fold Change values are derived from comparison of HDM treated versus untreated CD4 T cells from each group. (XLSX 60 kb) Additional file 11: Table S7. Transcription factor target enrichment of asthma-associated genes. We compared HDM driven responses in HDM-sensitized atopics with asthma versus HDM-sensitized atopics without asthma using differential network analysis. The resulting gene list was analysed using Enrichr showing that these asthma-associated genes were enriched with targets of STAT6 signaling. (XLS 29 kb)

## Abbreviations

DEG: Differentially expressed genes
HDM: House dust mite
PBMC: Peripheral blood mononuclear cells

## References

[1] Lambrecht BN, Hammad H (2015). The immunology of asthma. Nat Immunol.

[2] Lloyd CM, Hessel EM (2010). Functions of T cells in asthma: more than just T(H)2 cells. Nat Rev Immunol.

[3] Choy DF, Hart KM, Borthwick LA, Shikotra A, Nagarkar DR, Siddiqui S, Jia G, Ohri CM, Doran E, Vannella KM (2015). TH2 and TH17 inflammatory pathways are reciprocally regulated in asthma. Sci Transl Med.

[4] Hansen G, Berry G, DeKruyff RH, Umetsu DT (1999). Allergen-specific Th1 cells fail to counterbalance Th2 cell-induced airway hyperreactivity but cause severe airway inflammation. J Clin Invest.

[5] Ray A, Khare A, Krishnamoorthy N, Qi Z, Ray P (2010). Regulatory T cells in many flavors control asthma. Mucosal Immunol.

[6] Wills-Karp M (2004). Interleukin-13 in asthma pathogenesis. Immunol Rev.

[7] Busse WW, Morgan WJ, Gergen PJ, Mitchell HE, Gern JE, Liu AH, Gruchalla RS, Kattan M, Teach SJ, Pongracic JA (2011). Randomized trial of omalizumab (anti-IgE) for asthma in inner-city children. N Engl J Med.

[8] Wenzel S, Wilbraham D, Fuller R, Getz EB, Longphre M (2007). Effect of an interleukin-4 variant on late phase asthmatic response to allergen challenge in asthmatic patients: results of two phase 2a studies. Lancet.

[9] Hollams EM, Deverell M, Serralha M, Suriyaarachchi D, Parsons F, Zhang G, de Klerk N, Holt BJ, Ladyman C, Sadowska A (2009). Elucidation of asthma phenotypes in atopic teenagers through parallel immunophenotypic and clinical profiling. J Allergy Clin Immunol.

[10] Kramer A, Green J, Pollard J, Tugendreich S (2014). Causal analysis approaches in Ingenuity Pathway Analysis. Bioinformatics.

[11] Newnham JP, Evans SF, Michael CA, Stanley FJ, Landau LI (1993). Effects of frequent ultrasound during pregnancy: a randomised controlled trial. Lancet.

[12] Heaton T, Rowe J, Turner S, Aalberse RC, de Klerk N, Suriyaarachchi D, Serralha M, Holt BJ, Hollams E, Yerkovich S (2005). An immunoepidemiological approach to asthma: identification of in-vitro T-cell response patterns associated with different wheezing phenotypes in children. Lancet.

[13] Bosco A, McKenna KL, Devitt CJ, Firth MJ, Sly PD, Holt PG (2006). Identification of novel Th2-associated genes in T memory responses to allergens. J Immunol.

[14] Bosco A, McKenna KL, Firth MJ, Sly PD, Holt PG (2009). A network modeling approach to analysis of the Th2 memory responses underlying human atopic disease. J Immunol.

[15] Irizarry RA, Hobbs B, Collin F, Beazer-Barclay YD, Antonellis KJ, Scherf U, Speed TP (2003). Exploration, normalization, and summaries of high density oligonucleotide array probe level data. Biostatistics.

[16] Dai M, Wang P, Boyd AD, Kostov G, Athey B, Jones EG, Bunney WE, Myers RM, Speed TP, Akil H (2005). Evolving gene/transcript definitions significantly alter the interpretation of GeneChip data. Nucleic Acids Res.

[17] Kauffmann A, Gentleman R, Huber W (2009). arrayQualityMetrics--a bioconductor package for quality assessment of microarray data. Bioinformatics.

[18] Johnson WE, Li C, Rabinovic A (2007). Adjusting batch effects in microarray expression data using empirical Bayes methods. Biostatistics.

[19] Lu J, Kerns RT, Peddada SD, Bushel PR (2011). Principal component analysis-based filtering improves detection for Affymetrix gene expression arrays. Nucleic Acids Res.

[20] Piccolo SR, Sun Y, Campbell JD, Lenburg ME, Bild AH, Johnson WE (2012). A single-sample microarray normalization method to facilitate personalized-medicine workflows. Genomics.

[21] Talloen W, Hochreiter S, Bijnens L, Kasim A, Shkedy Z, Amaratunga D, Gohlmann H (2010). Filtering data from high-throughput experiments based on measurement reliability. Proc Natl Acad Sci U S A.

[22] Smyth GK (2004). Linear models and empirical bayes methods for assessing differential expression in microarray experiments. Stat Appl Genet Mol Biol.

[23] Mason MJ, Fan G, Plath K, Zhou Q, Horvath S (2009). Signed weighted gene co-expression network analysis of transcriptional regulation in murine embryonic stem cells. BMC Genomics.

[24] Bosco A, Ehteshami S, Panyala S, Martinez FD (2012). Interferon regulatory factor 7 is a major hub connecting interferon-mediated responses in virus-induced asthma exacerbations in vivo. J Allergy Clin Immunol.

[25] Fuller TF, Ghazalpour A, Aten JE, Drake TA, Lusis AJ, Horvath S (2007). Weighted gene coexpression network analysis strategies applied to mouse weight. Mamm Genome.

[26] Chen EY, Tan CM, Kou Y, Duan Q, Wang Z, Meirelles GV, Clark NR, Ma’ayan A (2013). Enrichr: interactive and collaborative HTML5 gene list enrichment analysis tool. BMC Bioinformatics.

[27] Yu G, Wang LG, Han Y, He QY (2012). clusterProfiler: an R package for comparing biological themes among gene clusters. Omics.

[28] Post S, Nawijn MC, Hackett TL, Baranowska M, Gras R, van Oosterhout AJ, Heijink IH (2012). The composition of house dust mite is critical for mucosal barrier dysfunction and allergic sensitisation. Thorax.

[29] Hammad H, Chieppa M, Perros F, Willart MA, Germain RN, Lambrecht BN (2009). House dust mite allergen induces asthma via Toll-like receptor 4 triggering of airway structural cells. Nat Med.

[30] Vaags AK, Bowdin S, Smith ML, Gilbert-Dussardier B, Brocke-Holmefjord KS, Sinopoli K, Gilles C, Haaland TB, Vincent-Delorme C, Lagrue E (2014). Absent CNKSR2 causes seizures and intellectual, attention, and language deficits. Ann Neurol.

[31] Cheyette BN, Waxman JS, Miller JR, Takemaru K, Sheldahl LC, Khlebtsova N, Fox EP, Earnest T, Moon RT (2002). Dapper, a Dishevelled-associated antagonist of beta-catenin and JNK signaling, is required for notochord formation. Dev Cell.

[32] Notani D, Gottimukkala KP, Jayani RS, Limaye AS, Damle MV, Mehta S, Purbey PK, Joseph J, Galande S (2010). Global regulator SATB1 recruits beta-catenin and regulates T(H)2 differentiation in Wnt-dependent manner. PLoS Biol.

[33] Taft RJ, Vanderver A, Leventer RJ, Damiani SA, Simons C, Grimmond SM, Miller D, Schmidt J, Lockhart PJ, Pope K (2013). Mutations in DARS cause hypomyelination with brain stem and spinal cord involvement and leg spasticity. Am J Hum Genet.

[34] Ma J, Cao X (2006). Regulation of Stat3 nuclear import by importin alpha5 and importin alpha7 via two different functional sequence elements. Cell Signal.

[35] Coste A, Antal MC, Chan S, Kastner P, Mark M, O’Malley BW, Auwerx J (2006). Absence of the steroid receptor coactivator-3 induces B-cell lymphoma. EMBO J.

[36] Yu C, York B, Wang S, Feng Q, Xu J, O’Malley BW (2007). An essential function of the SRC-3 coactivator in suppression of cytokine mRNA translation and inflammatory response. Mol Cell.

[37] Lund RJ, Loytomaki M, Naumanen T, Dixon C, Chen Z, Ahlfors H, Tuomela S, Tahvanainen J, Scheinin J, Henttinen T (2007). Genome-wide identification of novel genes involved in early Th1 and Th2 cell differentiation. J Immunol.

[38] Okamoto K, Iwai Y, Oh-Hora M, Yamamoto M, Morio T, Aoki K, Ohya K, Jetten AM, Akira S, Muta T (2010). IkappaBzeta regulates T(H)17 development by cooperating with ROR nuclear receptors. Nature.

[39] Mittal R, Patel K, Mittal J, Chan B, Yan D, Grati M, Liu XZ (2015). Association of PRPS1 Mutations with Disease Phenotypes. Dis Markers.

[40] Tang S, Chen T, Yu Z, Zhu X, Yang M, Xie B, Li N, Cao X, Wang J (2014). RasGRP3 limits Toll-like receptor-triggered inflammatory response in macrophages by activating Rap1 small GTPase. Nat Commun.

[41] Mashima R, Saeki K, Aki D, Minoda Y, Takaki H, Sanada T, Kobayashi T, Aburatani H, Yamanashi Y, Yoshimura A (2005). FLN29, a novel interferon- and LPS-inducible gene acting as a negative regulator of toll-like receptor signaling. J Biol Chem.

[42] Liao W, Schones DE, Oh J, Cui Y, Cui K, Roh TY, Zhao K, Leonard WJ (2008). Priming for T helper type 2 differentiation by interleukin 2-mediated induction of interleukin 4 receptor alpha-chain expression. Nat Immunol.

[43] Chen Q, Kim YC, Laurence A, Punkosdy GA, Shevach EM (2011). IL-2 controls the stability of Foxp3 expression in TGF-beta-induced Foxp3+ T cells in vivo. J Immunol.

[44] Boyman O, Sprent J (2012). The role of interleukin-2 during homeostasis and activation of the immune system. Nat Rev Immunol.

[45] Busse WW, Israel E, Nelson HS, Baker JW, Charous BL, Young DY, Vexler V, Shames RS, Daclizumab Asthma Study G (2008). Daclizumab improves asthma control in patients with moderate to severe persistent asthma: a randomized, controlled trial. Am J Respir Crit Care Med.

[46] Akdis CA (2012). Therapies for allergic inflammation: refining strategies to induce tolerance. Nat Med.

[47] Borish LC, Nelson HS, Lanz MJ, Claussen L, Whitmore JB, Agosti JM, Garrison L (1999). Interleukin-4 receptor in moderate atopic asthma. A phase I/II randomized, placebo-controlled trial. Am J Respir Crit Care Med.

[48] Mori A, Suko M, Kaminuma O, Inoue S, Ohmura T, Nishizaki Y, Nagahori T, Asakura Y, Hoshino A, Okumura Y (1996). IL-15 promotes cytokine production of human T helper cells. J Immunol.

[49] Ruckert R, Brandt K, Braun A, Hoymann HG, Herz U, Budagian V, Durkop H, Renz H, Bulfone-Paus S (2005). Blocking IL-15 prevents the induction of allergen-specific T cells and allergic inflammation in vivo. J Immunol.

[50] Bosco A, Ehteshami S, Stern DA, Martinez FD (2010). Decreased activation of inflammatory networks during acute asthma exacerbations is associated with chronic airflow obstruction. Mucosal Immunol.

[51] Laza-Stanca V, Message SD, Edwards MR, Parker HL, Zdrenghea MT, Kebadze T, Kon OM, Mallia P, Stanciu LA, Johnston SL (2011). The role of IL-15 deficiency in the pathogenesis of virus-induced asthma exacerbations. PLoS Pathog.

[52] Liu YJ (2007). Thymic stromal lymphopoietin and OX40 ligand pathway in the initiation of dendritic cell-mediated allergic inflammation. J Allergy Clin Immunol.

[53] Wenzel SE, Barnes PJ, Bleecker ER, Bousquet J, Busse W, Dahlen SE, Holgate ST, Meyers DA, Rabe KF, Antczak A (2009). A randomized, double-blind, placebo-controlled study of tumor necrosis factor-alpha blockade in severe persistent asthma. Am J Respir Crit Care Med.

[54] Dinarello CA (2011). Interleukin-1 in the pathogenesis and treatment of inflammatory diseases. Blood.

[55] Schmitz N, Kurrer M, Kopf M (2003). The IL-1 receptor 1 is critical for Th2 cell type airway immune responses in a mild but not in a more severe asthma model. Eur J Immunol.

[56] Huber JP, Ramos HJ, Gill MA, Farrar JD (2010). Cutting edge: Type I IFN reverses human Th2 commitment and stability by suppressing GATA3. J Immunol.

[57] Djukanovic R, Harrison T, Johnston SL, Gabbay F, Wark P, Thomson NC, Niven R, Singh D, Reddel HK, Davies DE (2014). The effect of inhaled IFN-beta on worsening of asthma symptoms caused by viral infections. A randomized trial. Am J Respir Crit Care Med.

[58] Akdis CA, Blesken T, Akdis M, Wuthrich B, Blaser K (1998). Role of interleukin 10 in specific immunotherapy. J Clin Invest.

[59] Francis JN, Till SJ, Durham SR (2003). Induction of IL-10 + CD4 + CD25+ T cells by grass pollen immunotherapy. J Allergy Clin Immunol.

[60] Zuany-Amorim C, Haile S, Leduc D, Dumarey C, Huerre M, Vargaftig BB, Pretolani M (1995). Interleukin-10 inhibits antigen-induced cellular recruitment into the airways of sensitized mice. J Clin Invest.

[61] Stampfli MR, Cwiartka M, Gajewska BU, Alvarez D, Ritz SA, Inman MD, Xing Z, Jordana M (1999). Interleukin-10 gene transfer to the airway regulates allergic mucosal sensitization in mice. Am J Respir Cell Mol Biol.

[62] Palomares O, Martin-Fontecha M, Lauener R, Traidl-Hoffmann C, Cavkaytar O, Akdis M, Akdis CA (2014). Regulatory T cells and immune regulation of allergic diseases: roles of IL-10 and TGF-beta. Genes Immun.

[63] Hawrylowicz CM, O’Garra A (2005). Potential role of interleukin-10-secreting regulatory T cells in allergy and asthma. Nat Rev Immunol.

[64] Pua HH, Ansel KM (2015). MicroRNA regulation of allergic inflammation and asthma. Curr Opin Immunol.

[65] Mattes J, Collison A, Plank M, Phipps S, Foster PS (2009). Antagonism of microRNA-126 suppresses the effector function of TH2 cells and the development of allergic airways disease. Proc Natl Acad Sci U S A.

[66] Collison A, Mattes J, Plank M, Foster PS (2011). Inhibition of house dust mite-induced allergic airways disease by antagonism of microRNA-145 is comparable to glucocorticoid treatment. J Allergy Clin Immunol.

[67] Simpson LJ, Patel S, Bhakta NR, Choy DF, Brightbill HD, Ren X, Wang Y, Pua HH, Baumjohann D, Montoya MM (2014). A microRNA upregulated in asthma airway T cells promotes TH2 cytokine production. Nat Immunol.

[68] Agarwal V, Bell GW, Nam JW, Bartel DP. Predicting effective microRNA target sites in mammalian mRNAs. eLife. 2015;4:e05005. 10.7554/eLife.05005 PMC4532895 26267216

[69] Gilani AH, Shah AJ, Ghayur MN, Majeed K (2005). Pharmacological basis for the use of turmeric in gastrointestinal and respiratory disorders. Life Sci.

[70] Subhashini, Chauhan PS, Kumari S, Kumar JP, Chawla R, Dash D, Singh M, Singh R (2013). Intranasal curcumin and its evaluation in murine model of asthma. Int Immunopharmacol.

[71] Oh SW, Cha JY, Jung JE, Chang BC, Kwon HJ, Lee BR, Kim DY (2011). Curcumin attenuates allergic airway inflammation and hyper-responsiveness in mice through NF-kappaB inhibition. J Ethnopharmacol.

[72] Maslowski KM, Mackay CR (2011). Diet, gut microbiota and immune responses. Nat Immunol.

[73] Maslowski KM, Vieira AT, Ng A, Kranich J, Sierro F, Yu D, Schilter HC, Rolph MS, Mackay F, Artis D (2009). Regulation of inflammatory responses by gut microbiota and chemoattractant receptor GPR43. Nature.

[74] Trompette A, Gollwitzer ES, Yadava K, Sichelstiel AK, Sprenger N, Ngom-Bru C, Blanchard C, Junt T, Nicod LP, Harris NL (2014). Gut microbiota metabolism of dietary fiber influences allergic airway disease and hematopoiesis. Nat Med.

[75] Skrindo I, Ballke C, Gran E, Johansen FE, Baekkevold ES, Jahnsen FL (2015). IL-5 production by resident mucosal allergen-specific T cells in an explant model of allergic rhinitis. Clin Exp Allergy.

[76] Gardeux V, Bosco A, Li J, Halonen MJ, Jackson D, Martinez FD, Lussier YA (2015). Towards a PBMC “virogram assay” for precision medicine: Concordance between ex vivo and in vivo viral infection transcriptomes. J Biomed Inform.

