# Supplementary figures and images for: Differential gene network analysis for the identification of asthma-associated therapeutic targets in allergen-specific T-helper memory responses
Source: BMC Med Genomics. 2016 Feb 27;9:9. doi: 10.1186/s12920-016-0171-z (PMC4769846; doi:10.1186/s12920-016-0171-z)

Additional Figure 1

Overlap of differentially expressed genes by group

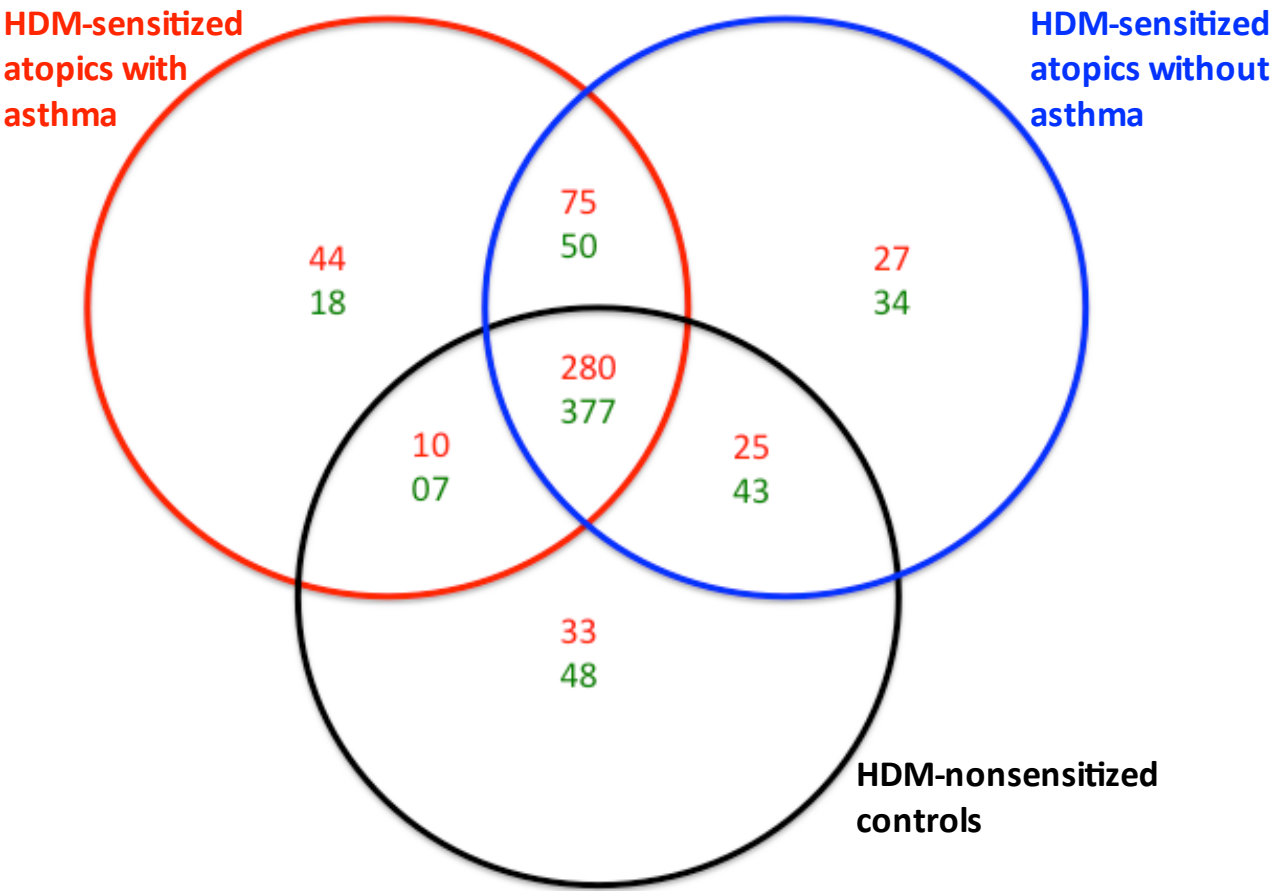

Supplement: Additional file 4: Figure S1. — Overlap of differentially expressed genes by group. Overlap of differentially expressed genes in HDM-sensitized atopics with asthma (red), HDM-sensitized atopics without asthma (blue) and non-sensitized controls (black). Gene expression patterns in CD4+ T cells were profiled by microarray and we compared HDM stimulated versus unstimulated CD4+ T cells for each group. Numbers in red indicate upregulated genes and numbers in green represent downregulated genes. (PDF 68 kb) [file 12920_2016_171_MOESM4_ESM.pdf]

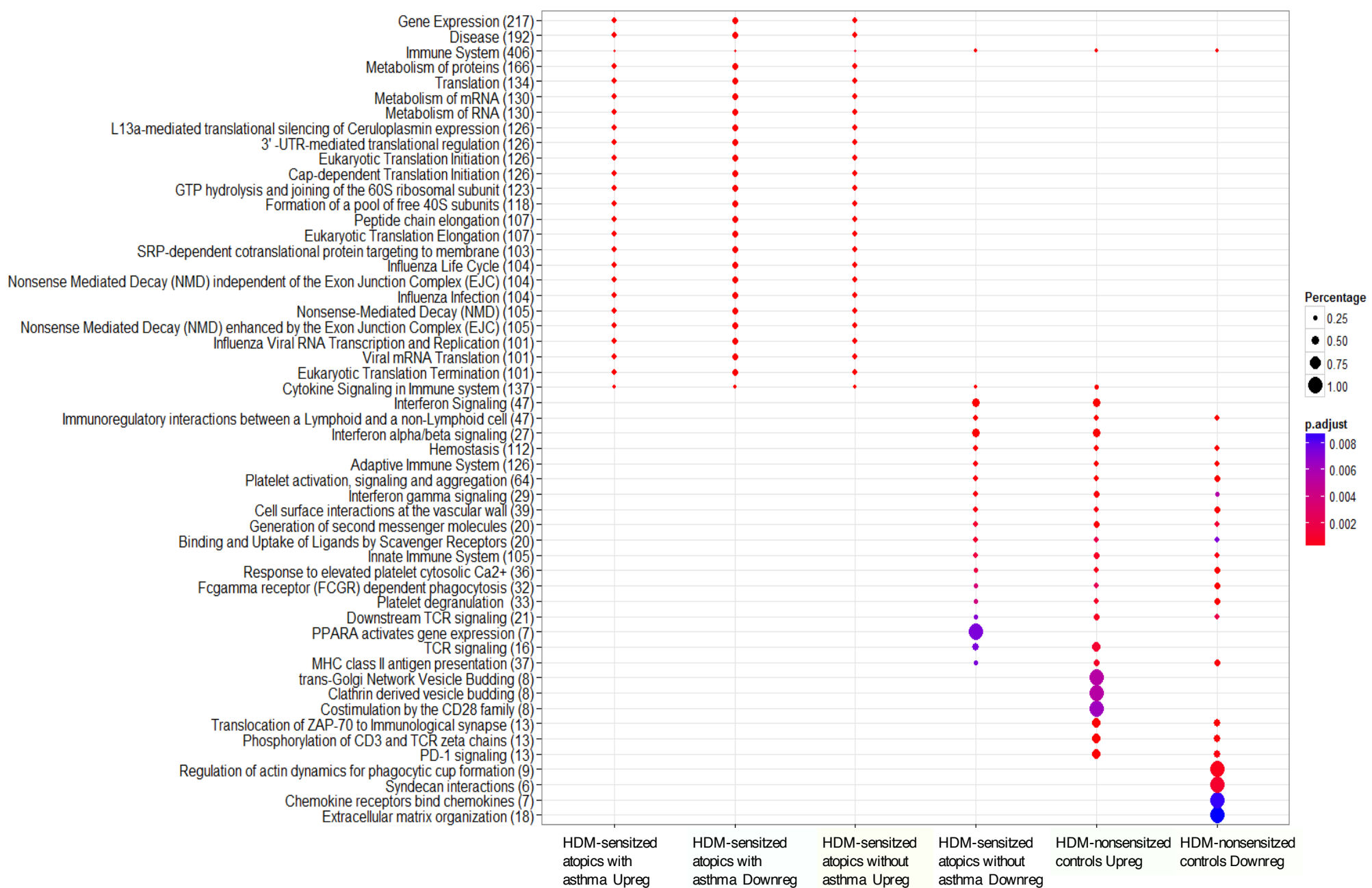

Supplement: Additional file 5: Figure S2. — Biological pathways enriched in the CD4 T cell responses to HDM from the three clinical groups. Data analysis by Cluster Profiler using reactome database. Biological pathways enriched in the set of upregulated (Upreg) and downregulated (Downreg) genes from HDM-stimulated versus resting CD4 T cells from HDM-sensitized atopics with/without asthma and HDM nonsensitized controls. (PDF 95 kb) [file 12920_2016_171_MOESM5_ESM.pdf]

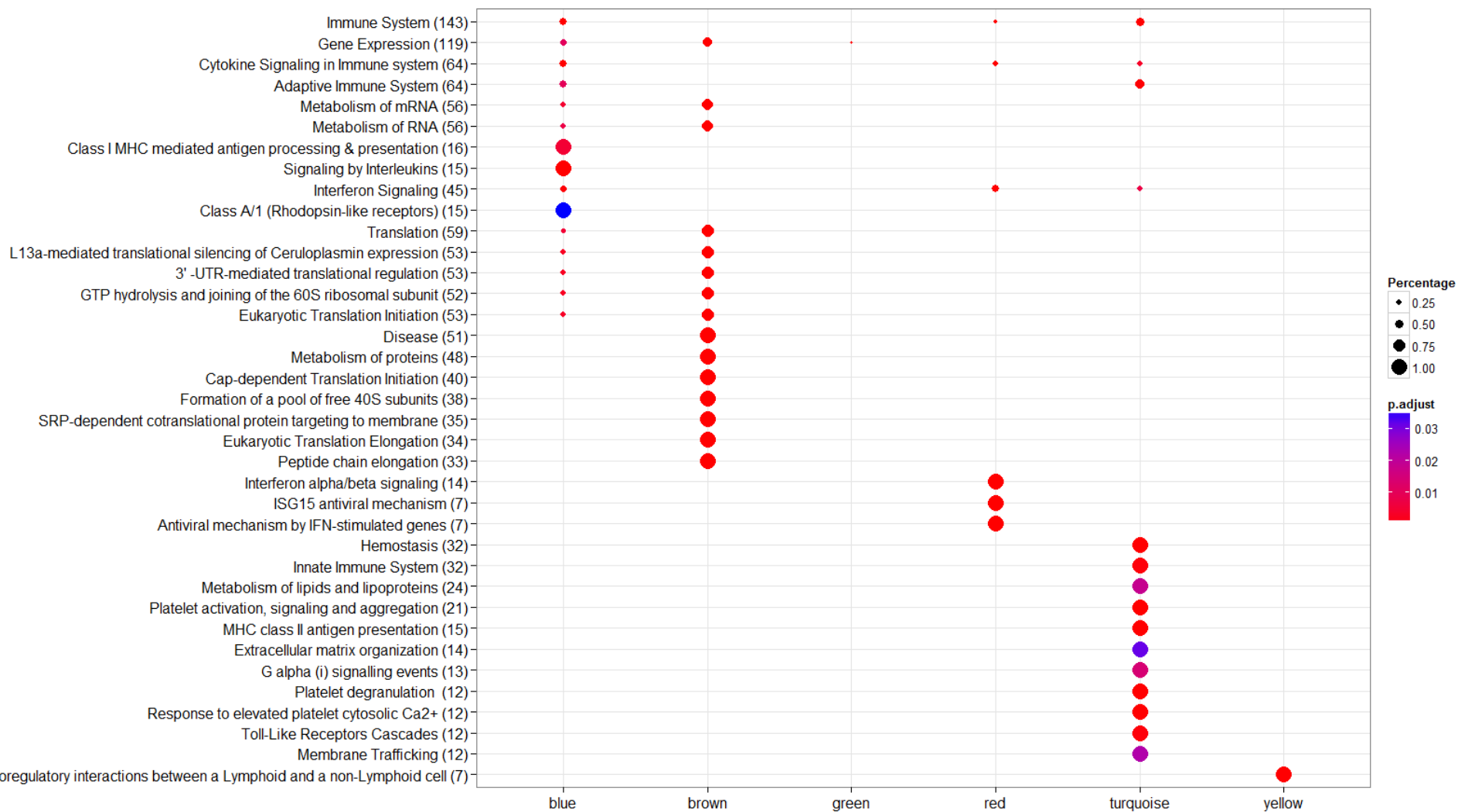

Supplement: Additional file 6: Figure S3. — Biological pathways enriched in the WGCNA modules in the CD4 T cell responses to HDM from HDM sensitized atopics with asthma. Data analysis by Cluster Profiler using reactome database. (PDF 73 kb) [file 12920_2016_171_MOESM6_ESM.pdf]

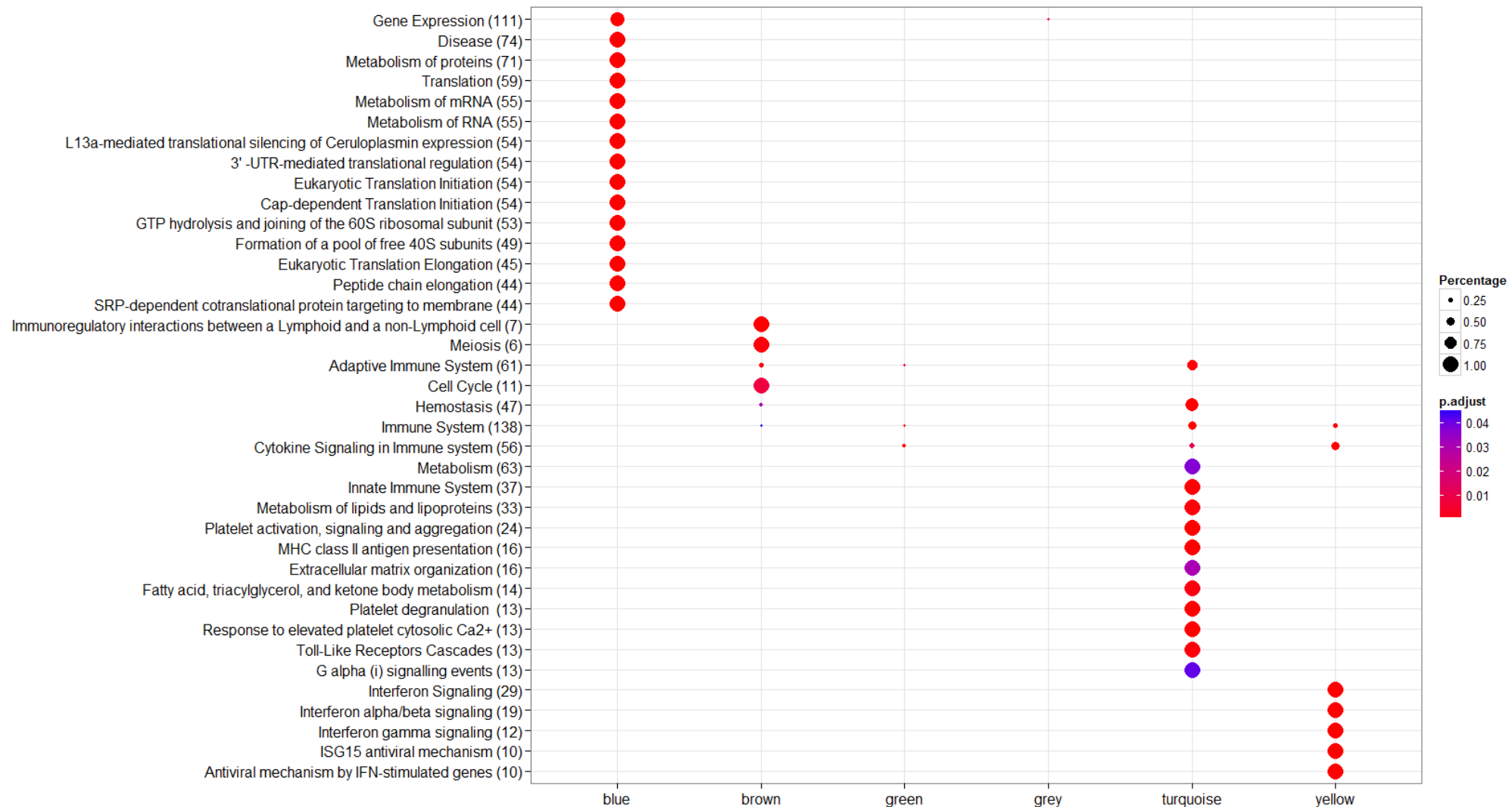

Supplement: Additional file 7: Figure S4. — Biological pathways enriched in the WGCNA modules in the CD4 T cell responses to HDM from HDM sensitized atopics without asthma. Data analysis by Cluster Profiler using reactome database. (PDF 72 kb) [file 12920_2016_171_MOESM7_ESM.pdf]
